# C-type Lectin Mincle Recognizes Glucosyl-diacylglycerol of *Streptococcus pneumoniae* and Plays a Protective Role in Pneumococcal Pneumonia

**DOI:** 10.1371/journal.ppat.1006038

**Published:** 2016-12-06

**Authors:** Friederike Behler-Janbeck, Tomotsugu Takano, Regina Maus, Jennifer Stolper, Danny Jonigk, Meritxell Tort Tarrés, Thomas Fuehner, Antje Prasse, Tobias Welte, Mattie S. M. Timmer, Bridget L. Stocker, Yoichi Nakanishi, Tomofumi Miyamoto, Sho Yamasaki, Ulrich A. Maus

**Affiliations:** 1 Department of Experimental Pneumology, Hannover Medical School, Hannover, Germany; 2 Division of Molecular Immunology, Medical Institute of Bioregulation, Kyushu University, Fukuoka, Japan; 3 Institute of Pathology, Hannover Medical School, Hannover, Germany; 4 Clinic for Pneumology, Hannover Medical School, Hannover, Germany; 5 German Center for Lung Research, partner site BREATH, Hannover, Germany; 6 School of Chemical and Physical Sciences, Victoria University of Wellington, Wellington, New Zealand; 7 Research Institute for Diseases of the Chest, Graduate School of Medical Sciences, Kyushu University, Fukuoka, Japan; 8 Department of Natural Products Chemistry, Graduate School of Pharmaceutical Sciences, Kyushu University, Fukuoka, Japan; The University of Alabama at Birmingham, UNITED STATES

## Abstract

Among various innate immune receptor families, the role of C-type lectin receptors (CLRs) in lung protective immunity against *Streptococcus pneumoniae* (*S*. *pneumoniae*) is not fully defined. We here show that Mincle gene expression was induced in alveolar macrophages and neutrophils in bronchoalveolar lavage fluids of mice and patients with pneumococcal pneumonia. Moreover, *S*. *pneumoniae* directly triggered Mincle reporter cell activation *in vitro* via its glycolipid glucosyl-diacylglycerol (Glc-DAG), which was identified as the ligand recognized by Mincle. Purified Glc-DAG triggered Mincle reporter cell activation and stimulated inflammatory cytokine release by human alveolar macrophages and alveolar macrophages from WT but not Mincle KO mice. Mincle deficiency led to increased bacterial loads and decreased survival together with strongly dysregulated cytokine responses in mice challenged with focal pneumonia inducing *S*. *pneumoniae*, all of which was normalized in Mincle KO mice reconstituted with a WT hematopoietic system. In conclusion, the Mincle-Glc-DAG axis is a hitherto unrecognized element of lung protective immunity against focal pneumonia induced by *S*. *pneumoniae*.

## Author Summary

Bacterial infections of the lung cause significant morbidity and mortality worldwide. The Gram-positive bacterium *Streptococcus pneumoniae* is the most prevalent pathogen causing bacterial lung infections in humans. Among the various innate immune receptors by which lung sentinel cells are able to sense bacterial invasion of the lung, relatively little knowledge exists regarding the role of C-type lectins in the process of bacterial recognition by the immune system. In this report, we show that *S*. *pneumoniae* is recognized by the C-type lectin receptor Mincle, which is a receptor specialized to bind pathogen-derived carbohydrate structures. In the current study, we identify a specific glycolipid of *S*. *pneumoniae* by which the bacterium is recognized by lung sentinel cells. Moreover, we show the critical importance of the described receptor-ligand interaction for survival of pneumococcal pneumonia in mice. Collectively, we identify the Mincle-Glc-DAG axis as an important element of lung host defense against focal pneumococcal pneumonia.

## Introduction

*Streptococcus pneumoniae* is the most prevalent pathogen causing community-acquired pneumonia (CAP). Pneumococcal CAP frequently progresses to invasive pneumococcal disease (IPD), which is associated with high morbidity and mortality rates worldwide [1–3].

Resident alveolar macrophages (AM) and neutrophils represent the first lines of host defense against lung-tropic bacterial pathogens, and have been characterized to express multiple pattern recognition receptors (PRRs), including Toll-like receptors (TLRs), NOD-like receptors (NLRs), and RIG-I-like receptors, as well as C-type lectin receptors (CLRs), all of which sense pathogen-associated molecular patterns (PAMPs) and/or danger-associated molecular patterns (DAMPs). Activation of PRRs leads to production of NF-ĸB-dependent proinflammatory cytokines such as TNF-α, IL-6, and IL-1β, followed by overlapping release of anti-inflammatory mediators, such as IL-1ra and IL-10, which together orchestrate and shape downstream lung antibacterial immune responses by recruitment and activation/de-activation of inflammatory leukocyte subsets [3–6]. However, dysregulated pro-/anti-inflammatory cytokine responses (‘cytokine storms’) have been recognized to contribute to severe lung tissue damage, which is typically observed in severe CAP (sCAP) [7, 8].

The macrophage-inducible C-type lectin receptor Mincle (also termed Clec4e or Clecsf9) is a type II transmembrane C-type lectin, which is strongly induced in response to inflammatory stimuli, such as LPS, TNF-α, IL-6 or IFN-γ, and cellular stress [9, 10]. Mincle is expressed on myeloid cells including macrophages, dendritic cells, neutrophils, but also on B cells, but not on NK cells [10–12]. In its transmembrane region, Mincle has a positively charged arginine residue [10] and is associated with an ITAM-containing adaptor molecule Fc receptor common γ chain (FcRγ) through charge-charge interaction to transduce activating signals into the cell [10, 13]. Ligand binding to Mincle leads to phosphorylation of ITAM in the FcRγ chain and downstream recruitment of Syk kinase, followed by a heterotypic aggregation of Card9 with Bcl10 and Malt1. This signaling pathway results in an adaptive Th1 and Th17 cytokine-dominated immune response and triggers production of cytokines such as TNF-α, IL-6, MIP-2, IFN-γ and IL-17 [13–15]. Mincle has been identified as sensor for the mycobacterial cell wall component trehalose-6,6’-dimycolate (TDM, Cord Factor) as well as its synthetic derivative trehalose-6,6’-dibehenate (TDB) [15, 16]. Molecules with a similar structure like TDM are also reported to be Mincle ligands. For example, human Mincle recognizes glycerol monocorynomycolate derived from mycobacteria [17]. Brartemicin derived from actinomycetes binds to human and bovine Mincle [18]. Except for molecules having a similar structure with TDM, mannosyl fatty acids and β-gentiobiosyl glyceroglycolipids derived from *Malassezia* spp. are also reported as Mincle ligands [19]. Additionally, Mincle is induced in response to the pathogenic fungi *Candida albicans* as well as *Malassezia* spp. [19–21]. We recently demonstrated that Mincle is expressed on AM, newly recruited exudate macrophages and alveolar recruited neutrophils in response to *Myocobacterium bovis* (*M*. *bovis*) BCG infection, where it critically shaped the lung inflammatory response after mycobacterial challenge, and contributed to control of extrapulmonary *M*. *bovis* BCG infection in mice [22, 23].

Lung infections with *S*. *pneumoniae* usually manifest as lobar pneumonia either or not progressing to invasive pneumococcal disease (IPD), partially depending on the serotypes involved [24]. To mimic these different disease courses, in the current study, we used different serotypes of *S*. *pneumoniae* either causing focal pneumonia in the absence of bacteremia (type 19F *S*. *pneumoniae*), or invasive serotype 3 *S*. *pneumoniae* rapidly progressing to IPD [25, 26]. We here report that Mincle is specifically important to lung protective immunity against lobar pneumonia but not IPD.

## Results

### *S*. *pneumoniae* triggers expression of Mincle in the lungs of mice and patients with pneumococcal pneumonia

**Fig 1:**
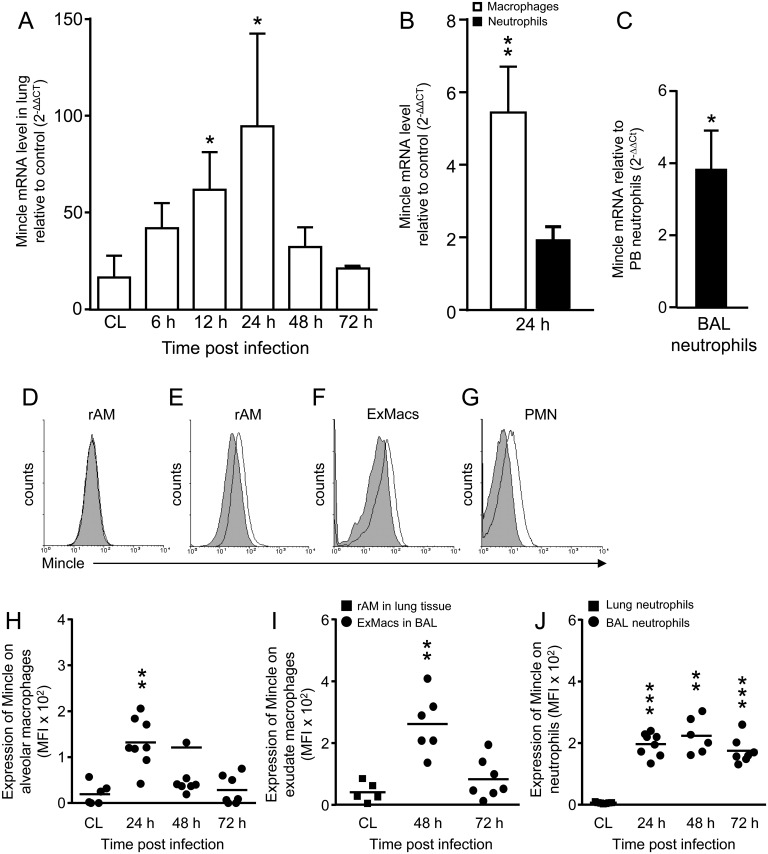
Expression of Mincle in the lungs of mice after infection with *S*. *pneumoniae*. (A) WT mice were infected with *S*. *pneumoniae* (1 x 10^7^ CFU/mouse) or were mock-infected (PBS), and lungs were harvested at the given time points and Mincle mRNA levels were analyzed. Values are shown as mean ± SD (n = 3 mice per time point and treatment group). CL (control): Mock-infection relative to untreated control, all other values in (A), relative to mock-infection. * p<0.05, relative to mock-infection. (B) Flow sorted alveolar macrophages and neutrophils purified from lung tissue of mock- versus *S*. *pneumoniae*-infected WT mice were subjected to Mincle mRNA analysis by real-time RT-PCR. Data are shown as mean ± SD (n = 4–6 mice for mock-infected and n = 5 mice for *S*. *pneumoniae*-infected mice). * p<0.05 relative to neutrophils. (C) Mincle mRNA levels in neutrophils collected by bronchoalveolar lavage from the lungs of patients with pneumococcal pneumonia, relative to peripheral blood neutrophils collected from the same patients. Data are shown as mean ± SD (n = 3 patients). * p<0.05 relative to PB neutrophils. (D-J) WT mice were left untreated (D, and CL in H-J), or were infected with *S*. *pneumoniae* (10^6^ CFU/mouse). At 0 h, 24 h, 48 h, and 72 h post-infection, expression of Mincle was analyzed on BAL AM (D (0 h), E (24 h), and H), and BAL ExMacs (F (48 h), and I), and BAL neutrophils (G (48 h), and J) (see also S1 Fig). Note that CL in (I,J) represents Mincle expression on resident alveolar macrophages (I) and neutrophils (J) purified from lung tissue of mock-infected WT mice. Horizontal bars: median values (n = 5–8 mice). Experiments were repeated two times with similar results. ** p<0.01, *** p<0.001, relative to mock-infection ((Mann-Whitney U test). ExMacs, exudate macrophages.

In initial experiments, we assessed the expression of C-type lectin Mincle in WT mice infected with focal pneumonia inducing serotype 19F *S*. *pneumoniae*. As shown in Fig 1A, Mincle gene expression was found to peak in lung tissue of mice at 24 h post *S*. *pneumoniae* challenge, with a strong decline towards 72 h post-infection. Moreover, Mincle gene expression was significantly increased in alveolar macrophages and neutrophils of mice at 24 h post *S*. *pneumoniae* infection (Fig 1B). Similarly, we found that neutrophils collected by bronchoalveolar lavage from the lungs of patients with confirmed pneumococcal pneumonia also exhibited significantly upregulated Mincle gene expression, relative to peripheral blood neutrophils of the same patients (Fig 1C), demonstrating similar induction of Mincle gene expression in mice and humans in response to pneumococcal pneumonia.

We next examined cell surface expression of Mincle on resident AM and alveolar recruited exudate macrophages (ExMacs, S1 Fig) and neutrophils in BAL fluids of *S*. *pneumoniae* infected WT mice. As shown in Fig 1, we found increased Mincle cell surface expression on alveolar macrophages at 24 h post-infection, and a decline towards baseline levels by 72 h post-infection (Fig 1E and 1H), relative to Mincle expression on resident AM from mock-infected mice (D, CL in H). BAL fluid exudate macrophages of *S*. *pneumoniae* infected mice upregulated Mincle on their cell surface at 48 h post-challenge, with decline towards baseline levels at 72 h post-infection (Fig 1F and 1I). Similarly, lung neutrophils from mock-infected WT mice demonstrated very low Mincle expression on their cell surface, which was sustained upregulated on alveolar recruited neutrophils at 24 h until 72 h post-infection (Fig 1G and 1J).

### Mincle senses *S*. *pneumoniae* via its glycolipid glucosyl-diacylglycerol (Glc-DAG)

**Fig 2:**
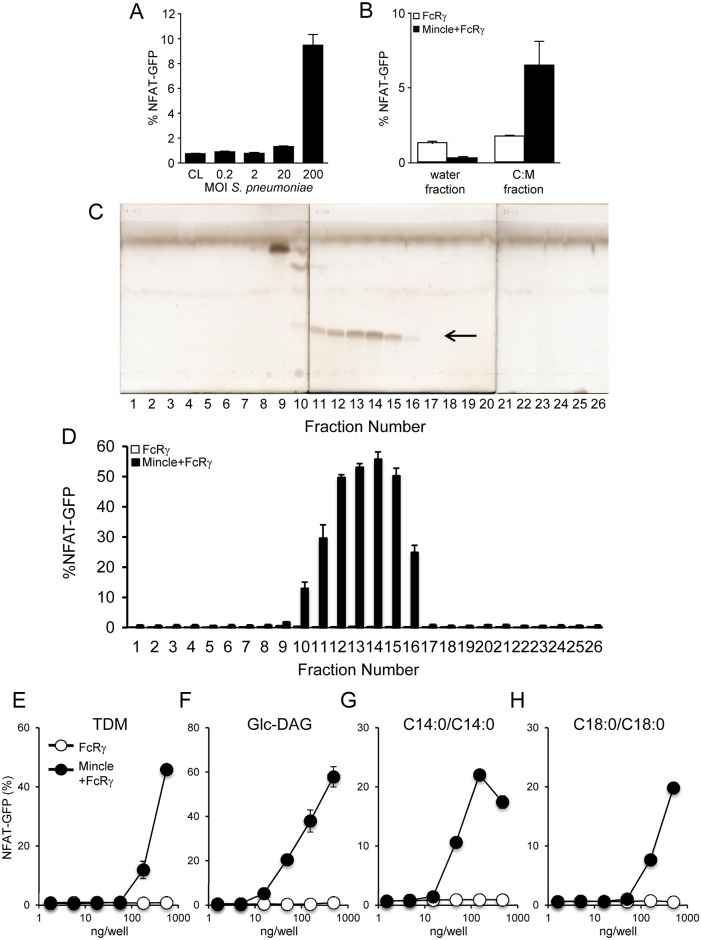
Characterization of glycolipid Glc-DAG purified from *S*. *pneumoniae*. (A) NFAT-GFP reporter cells (4 x 10^4^ cells/well) were incubated with live *S*. *pneumoniae* at MOI 0.2, 2, 20 and 200 for 18 h followed by determination of the percentage of GFP-expressing reporter cells by flow cytometry. (B) FcRγ or Mincle+FcRγ expressing NFAT-GFP reporter cells (4 x 10^4^ cells/well) were incubated with aqueous or C:M fraction of *S*. *pneumoniae* lysates for 18 h, followed by determination of GFP-expressing reporter cells by flow cytometry. (C) HPTLC result of HPLC fractionated C:M portion of pneumococcal lysates with putative ligand indicated by an arrow head (copper acetate stain). (D) FcRγ (white bars) or Mincle + FcRγ (black bars) NFAT-GFP reporter cell assay of HPLC fractions obtained from scratched and purified HPTLC bands of *S*. *pneumoniae* lysates shown in (C). Experiments were repeated three times with similar results. See also S2 Fig. (E-H) Effect of TDM (E), or *S*. *pneumoniae*-derived Glc-DAG (F), or synthetic Glc-DAG (C14:0/C14:0, (G), or C18:0/C18:0, (H)) to trigger GFP reporter activation in Mincle/FcRγ (black dots), or FcRγ only (white dots) expressing NFAT-GFP reporter cells.

The next set of experiments aimed to determine whether *S*. *pneumoniae* would be recognized by Mincle through direct receptor-ligand interaction. Indeed, stimulation of NFAT-GFP reporter cells with *S*. *pneumoniae* resulted in strong reporter cell GFP fluorescence emission (Fig 2A). Subsequent fractionation of pneumococcal lysates into an aqueous and organic (chloroform:methanol, C:M) phase and subsequent analysis of these fractions in our reporter cell assay revealed reporter cell activity only in the C:M but not aqueous fractions, implying that the putative ligand is contained in the organic phase of pneumococcal lysates (Fig 2B). Subsequent fractionation of lipid extracts of *S*. *pneumoniae* by high performance liquid chromatography (HPLC) revealed sub-fractions triggering a strong reporter cell activation (Fig 2C and 2D). We then purified these bands from sub-fractions with HPTLC. To identify the structure of these lipids, we performed electrospray ionization-mass spectrometry (ESI-MS), which showed four major peaks with mass-to-charge ratios of 697.4875, 725.5135, 751.5316, and 779.5608, which gave the molecular formula C_37_H_70_ NaO_10_, C_39_H_74_NaO_10_, C_41_H_76_NaO_10_ and C_43_H_80_NaO_10_ (calculated for 697.4861, 725.5174, 751.5316, and 779.5644, respectively) (S2A Fig). Composition of the glycolipid ligand was determined with GC-MS after acid hydrolysis using HCl/MeOH. The GC-MS chromatogram of FAMEs showed six FAMEs components and the major peak corresponded to methyl palmitate (16:0) (S2B and S2C Fig). The GC-MS chromatogram of methanol layer after TMS derivatization showed peaks due to TMS-glycerol (6.3 min) and TMS-glucose (13.9 and 14.1 min), respectively (S2D Fig). From these data, we conclude that the ligand is glucosyl-diacylglycerol (Glc-DAG), which contains various combinations of mainly C16:0 fatty acids binding to the glycerol backbone. Saturated palmitic acid was identified as the major fatty acid binding to the glycerol backbone, while various other saturated and unsaturated fatty acids were also contained in Glc-DAG (S1 Table). Importantly, similar to TDM serving as prototypic Mincle ligand (Fig 2E) and pneumococcus-derived Glc-DAG (Fig 2F), synthetic Glc-DAG (Fig 2G and 2H) also triggered Mincle reporter cell activation. Together, these data for the first time identify *S*. *pneumoniae*-derived Glc-DAG (S2E Fig) as a novel ligand of C-type lectin Mincle.

### Effect of Glc-DAG on inflammatory cytokine responses in vitro

We initially verified that Glc-DAG preparations were not contaminated with TLR4 ligands such as lipopolysaccharide (LPS). TLR4 reporter cells responded to stimulation with LPS but not to stimulation with Glc-DAG (S3A Fig). Moreover, we found that bone marrow-derived phagocytes from WT but not Myd88 KO mice responded to LPS stimulation with significantly increased TNF-α and MIP-2 cytokine release, whereas both WT and Myd88-deficient cells showed a similar TNF-α and MIP-2 cytokine release after Glc-DAG stimulation (S3B and S3C Fig), collectively confirming that Glc-DAG preparations did not contain any contaminations signaling via TLR4 or Myd88-dependent pathways.

**Fig 3:**
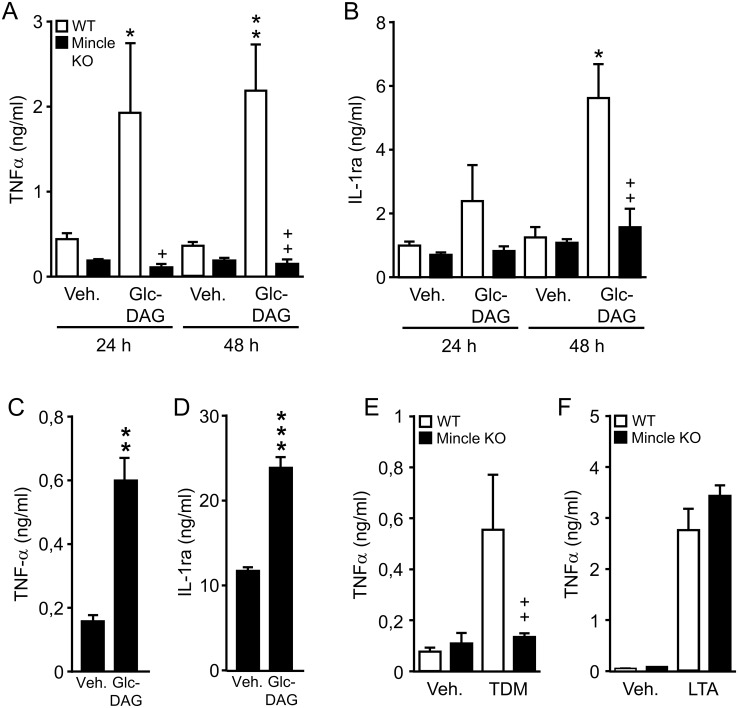
Mincle is an essential receptor for Glc-DAG purified from *S*. *pneumoniae*. (A,B) AM were collected from the lungs of untreated WT and Mincle KO mice, or were collected by bronchoalveolar lavage from healthy volunteers (C,D). After adherence purification, murine or human AM were seeded into vehicle (isopropanol), or Glc-DAG coated (5 μg/well), or TDM coated (1 μg/well) 96-well plates (7.5 x 10^4^ cells/well), or were stimulated with LTA (dissolved in sterile water) at 1 μg/ml, as indicated. After 24 and 48 h (A,B), or after 24 h (C-F), murine (A,B, E,F) or human (C,D) TNF-α or IL-1ra release was determined in cell culture supernatants by ELISA. Data are shown as mean ± SD of 4 determinations per time point and treatment condition and the data are representative of two independent experiments. In (C,D), cytokine values are depicted as mean ± SD of quadruplicate determinations per time point and treatment condition collected from human AM of one healthy individual, with similar data obtained from two other healthy individuals. *p<0.05, **p<0.01 compared to vehicle treated AM and +p<0.05, ++p<0.01 compared to AM from WT mice (unpaired t-test).

We then determined the effect of Glc-DAG on cytokine liberation by freshly isolated resident AM of WT and Mincle KO mice, as well as resident AM collected from healthy human volunteers *in vitro*. As shown in Fig 3A and 3B, stimulation of mouse AM with Glc-DAG significantly increased proinflammatory TNF-α and anti-inflammatory IL-1ra protein release in cultures of WT but not Mincle KO macrophages, at both 24 h and 48 h post-stimulation, and a similar TNF-α and IL-1ra cytokine response was also observed in cultures of Glc-DAG stimulated human AM (Fig 3C and 3D; 24 h post-stimulation). As expected, TDM representing a prototypic Mincle ligand also stimulated TNF-α release by WT but not Mincle KO alveolar macrophages, while LTA as TLR2 ligand not signaling via Mincle [19] triggered TNF-α release by both WT and Mincle KO macrophages (Fig 3E and 3F).

We next analyzed whether Glc-DAG purified from *S*. *pneumoniae* would directly provoke anti-bacterial respiratory burst formation in primary AM or neutrophils from WT as compared to Mincle KO mice. However, as shown in S4 Fig, Glc-DAG did not cause any burst induction in AM or neutrophils from WT or Mincle KO mice, whereas live *S*. *pneumoniae* triggered a comparable burst induction in both primary AM and neutrophils of both WT and Mincle KO mice *in vitro*. These data show that *S*. *pneumoniae* induced respiratory burst in AM or neutrophils is independent of the Mincle-Glc-DAG axis.

### Effect of Mincle deficiency on outcome of mice infected with invasive serotype 3 as compared to focal pneumonia inducing serotype 19F *S*. *pneumoniae*

**Fig 4:**
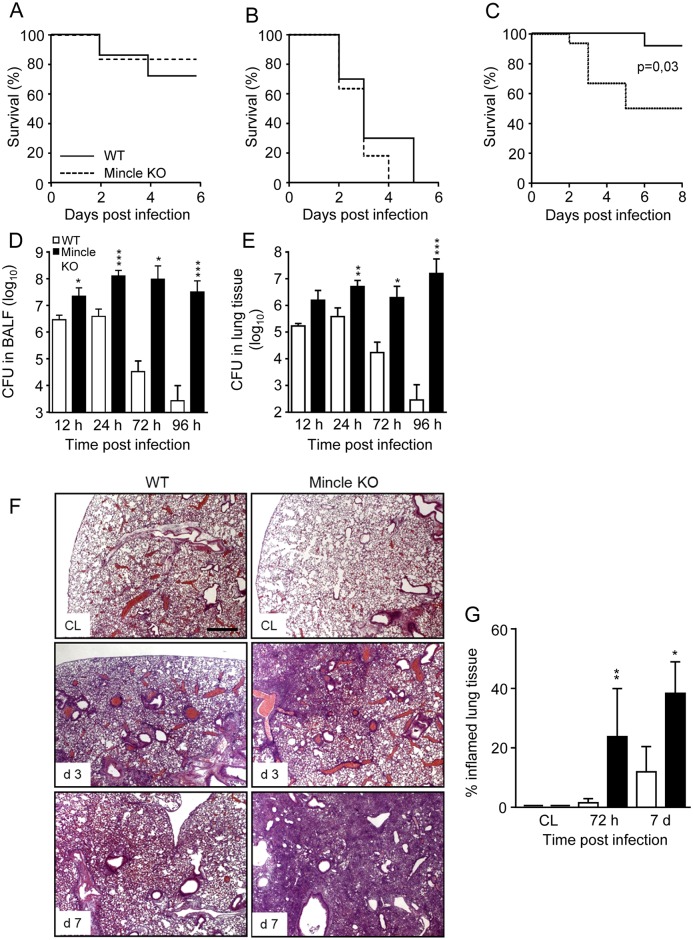
Outcome in WT and Mincle KO mice challenged with *S*. *pneumoniae*. (A,B) WT mice (solid lines) and Mincle KO mice (dashed lines) were infected with either highly invasive serotype 3 *S*. *pneumoniae* (A66.1; 2 x 10^5^ (A), or 2 x 10^6^ CFU/mouse, B), or were infected with focal pneumonia causing serotype 19F *S*. *pneumoniae* (C) (EF3030; 10^7^ CFU/mouse), and survival was monitored during an observation period of 6 or 8 days, as indicated (n = 10 mice per group). The data are representative of two independently performed experiments. (D,E) WT and Mincle KO mice were infected with serotype 19F *S*. *pneumoniae* (10^7^ CFU/mouse), and at indicated time points, bacterial loads were determined in BAL fluids (D) and lung tissue (E). Values are shown as mean ± SD of n = 5–8 mice per time point and treatment group. Data are representative of three independent experiments with similar results. *p<0.05, **p<0.01, ***p>0.001 relative to WT mice. (Mann-Whitney U test). (F) WT mice and Mincle KO mice were either left untreated or were infected with serotype 19F *S*. *pneumoniae* (10^7^ CFU/mouse). Lungs were harvested and lung tissue sections were stained with hematoxylin/eosin and the percentage of inflamed lung tissue was determined, as indicated in (G). Photographs were taken at 2.5 x original magnification. Scale bar, 500 μm. Data are shown as mean ± SD of n = 4–5 mice per time point and treatment group with *p<0.05, **p<0.01 relative to WT mice. (Mann-Whitney U test).

We next examined survival of WT and Mincle KO mice in two infection models reflecting the main clinical phenotypes of pneumococcal lung infections, i.e., invasive pneumococcal disease after infection with highly invasive serotype 3 *S*. *pneumoniae*, or focal pneumococcal pneumonia in the absence of bacteremia after infection with serotype 19F *S*. *pneumoniae* [25, 27]. As shown in Fig 4A and 4B, both WT mice and Mincle KO mice similarly succumbed to IPD after infection with either 2x10^5^ CFU/mouse (Fig 4A), or 2 x 10^6^ CFU/mouse (Fig 4B) of highly invasive serotype 3 *S*. *pneumoniae*, consistent with recent reports [26, 28]. In contrast, infection of mice with focal pneumonia-inducing serotype 19F *S*. *pneumoniae* caused an overall mortality of just 10% in WT mice during an observation period of 8 days while Mincle KO mice demonstrated a significantly increased mortality of ~50% post-infection (Fig 4C). Consistent with such increased mortality, Mincle KO mice demonstrated pneumococcal outgrowth in lung distal airspaces, whereas WT mice were able to purge bacteria in lung distal airspaces by day 4 post-challenge (Fig 4D and 4E).

Microscopic examination of lung tissue sections of mock-infected WT versus Mincle KO mice revealed normal lung architecture with regular bronchiolar and alveolar structures in either experimental group (Fig 4F, CL). After type 19F *S*. *pneumoniae* infection, we observed purulent, mostly focal bronchopneumonia with accompanying organizing pneumonia in lung tissue sections of Mincle KO mice on day 3 and even more so on day 7 post-infection, which was significantly less pronounced in WT mice (Fig 4F and 4G), collectively demonstrating that Mincle is particularly important to lung protective immunity against focal pneumonia-causing *S*. *pneumoniae* in mice.

### Effect of Mincle deficiency on lung leukocyte recruitment and proinflammatory cytokine release in mice infected with *S*. *pneumoniae*

Based on the observation that Mincle KO mice had severe defects in purging bacterial loads in their lungs, we next examined inflammatory lung leukocyte recruitment in WT and Mincle KO mice during pneumococcal pneumonia. As shown in S5 Fig, we did not observe any significant differences in either numbers of resident AM (S5A Fig), or newly recruited exudate macrophages (S5B Fig), or alveolar recruited neutrophils in BAL fluids of WT versus Mincle KO mice until 72 h post-infection, except that just on day 4 post-challenge, Mincle KO mice had slightly but significantly increased neutrophil counts in their BAL fluids relative to *S*. *pneumoniae* infected WT mice (S5C Fig). Collectively, these data illustrate that differences in mortality between groups were not due to differences in inflammatory lung leukocyte recruitment during pneumococcal pneumonia.

**Fig 5:**
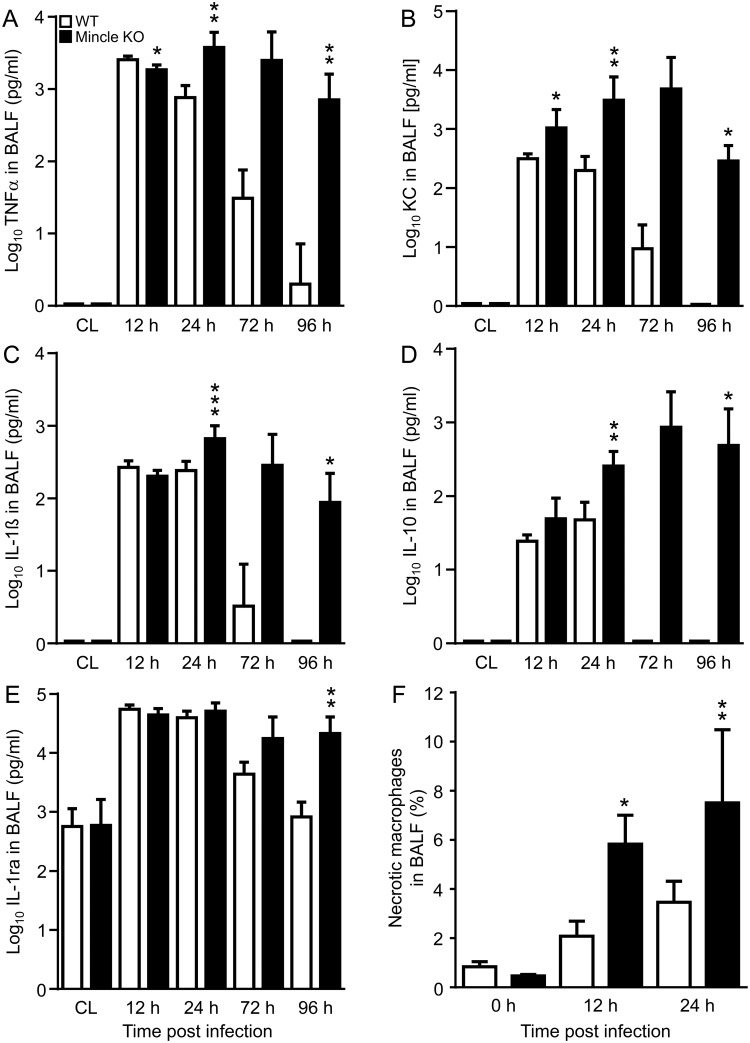
Impact of Mincle deficiency on lung proinflammatory cytokine release and macrophage necrosis in mice infected with *S*. *pneumoniae*. WT and Mincle KO mice were mock-infected or infected with *S*. *pneumoniae* (10^7^ CFU/mouse), and at indicated time points post-infection, BAL fluid TNF-α (A), KC (B), IL-1β (C), IL-10 (D), and IL-1ra (E) cytokine levels were measured. Data are shown as mean ± SD of n = 5–8 mice per time point and treatment group (12 h, n = 4 mice). Data are representative of two independent experiments with similar results. (F) WT and Mincle KO mice were mock-infected or low-dose infected with *S*. *pneumoniae* (5 x 10^5^ CFU/mouse). At the indicated time points, the percentage of necrotic macrophages (propidium iodide^pos^/annexin V^neg^) in BAL fluid was determined by flow cytometry. Values are shown as mean ± SD with n = 3 (0 h values) or 8 mice (12 and 24 h values) per time point and treatment group and are representative of two experiments. *p<0.05, **p<0.01, ***p<0.001 relative to WT mice. (Mann-Whitney U test).

Early proinflammatory and later developing anti-inflammatory cytokine responses are critical for orchestrating lung leukocyte recruitment and activation in response to lung bacterial infection. However, overwhelming ‘cytokine storms’ may be fatal for the outcome of severe bacterial pneumonia [2, 7]. Based on differences in bacterial loads and lung histopathology between WT and Mincle KO mice, we measured pro- and anti-inflammatory cytokine release in WT and KO mice challenged with type 19F *S*. *pneumoniae*. WT mice exhibited tightly regulated waves of pro- and anti-inflammatory cytokine release peaking at 12 h-24 h with a decline to baseline levels by 96 h post-infection. In contrast, Mincle KO mice responded with a severely disturbed pro-/anti-inflammatory cytokine response with sustained increased secretion of proinflammatory cytokines TNF-α (Fig 5A), KC (Fig 5B), and IL-1β (Fig 5C), accompanied by sustained release of anti-inflammatory cytokines IL-10 (Fig 5D) and IL-1ra (Fig 5E) during the observation period of 4 days. Moreover, Mincle KO mice responded with significantly increased alveolar macrophage necrosis to infection with type 19F *S*. *pneumoniae*, relative to control mice (Fig 5F).

Since Mincle KO mice had increased bacterial loads in their lungs after pneumococcal infection, we questioned whether lack of Mincle would affect macrophage or neutrophil phagocytosis and killing of *S*. *pneumoniae in vitro*. As shown in S6 Fig, resident AM and bone marrow-derived neutrophils from WT and Mincle KO mice were equally capable of phagocytosing *S*. *pneumoniae* (30 min value in S6A and S6B Fig). Similarly, the capacity of AM or neutrophils to kill intracellular bacteria at 90 and 120 minutes post-infection was comparable between groups, demonstrating that basic antibacterial features of AM and neutrophils including oxidative burst (S4 Fig), phagocytosis and killing of pneumococci (S6 Fig) is not dependent on the presence of Mincle.

### Reconstitution of Mincle KO mice with the hematopoietic system of WT mice restores their bacterial killing defects

**Fig 6:**
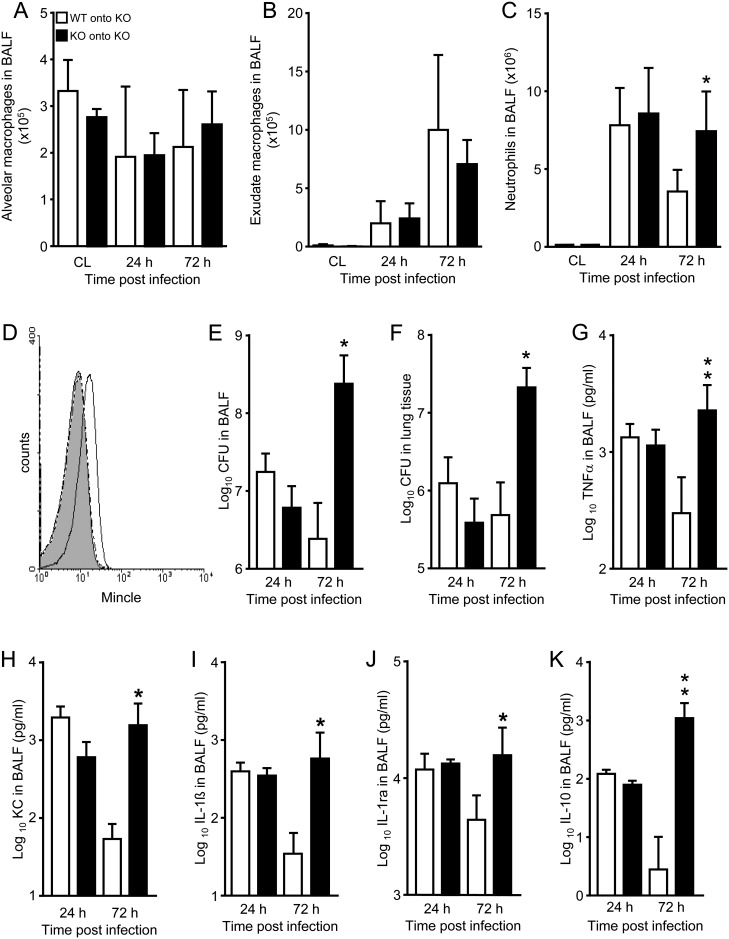
Lung anti-pneumococcal immunity is restored in chimeric Mincle KO mice reconstituted with the hematopoietic system of WT mice. Mincle KO mice received whole body irradiation (8 Gy), followed by transplantation with bone marrow cells (10^7^ cells/mouse i.v.) from either WT mice (WT onto KO, white bars), or Mincle KO mice (KO onto KO, transplantation controls, black bars). Seven weeks later, mice were infected orotracheally with 10^7^ CFU/mouse of type 19F *S*. *pneumoniae*. (A-C) Determination of BAL fluid cellular constituents (A, alveolar macrophages; B, alveolar exudate macrophages; C, alveolar recruited neutrophils) under baseline conditions (CL), or in response to infection, as indicated. (D) Analysis of Mincle expression on the cell surface of alveolar recruited neutrophils in bronchoalveolar lavage from *S*. *pneumoniae*-infected (24 h) Mincle KO mice reconstituted with WT (solid lines) or Mincle KO bone marrow cells (dashed lines). Grey histogram, isotype-stained negative control. (E,F) Determination of CFU counts in BAL fluids (E) or lung tissue (F), as indicated. (G-K) BAL fluid levels of proinflammatory cytokines TNFα (G), KC (H), IL-1β (I), or anti-inflammatory cytokines IL1ra (J), and IL-10 (K) in WT onto KO mice, or KO onto KO mice, as indicated. The data are shown as mean ± SD of n = 5 mice (A-F) or n = 4 mice (G-K) per time point and treatment group, and are representative of two experiments (Mann-Whitney U test).

Since Mincle KO mice demonstrated significantly increased bacterial loads in their lungs after infection with serotype 19F *S*. *pneumoniae*, we examined whether alveolar immigration of Mincle expressing effector cells would improve the attenuated antibacterial response in KO mice. Therefore, we reconstituted Mincle KO mice with the hematopoietic system of WT mice or Mincle KO mice serving as transplantation controls, thus allowing us to dissect the function of alveolar immigrating cells from that of alveolar residential cells in terms of lung protection against *S*. *pneumoniae*. Under baseline conditions, we found similar numbers of alveolar macrophages but no exudate macrophages or neutrophils in BAL fluids of WT onto Mincle KO mice, relative to Mincle KO onto Mincle KO mice (CL in Fig 6A–6C). In response to *S*. *pneumoniae* infection, no major differences in cell counts of alveolar macrophages, newly recruited exudate macrophages and neutrophils were noted between groups, except for neutrophils that were significantly increased in BAL fluids of KO onto KO mice at 72 h post-infection (Fig 6A–6C), similar to the findings made in Spn-infected Mincle KO mice (S5C Fig). As expected, neutrophils represented the predominant leukocyte subset in BAL fluids of *S*. *pneumoniae* infected KO onto KO and WT onto KO mice at 24 h post-infection (>96%), while only alveolar recruited neutrophils from WT onto KO, but not KO onto KO mice were found to express Mincle on their cell surface (Fig 6D). Moreover, WT onto Mincle KO mice demonstrated normal purging of pneumococci in their lungs, whereas KO onto KO mice demonstrated pneumococcal outgrowth in their lungs (Fig 6E and 6F), similar to Mincle KO mice infected with *S*. *pneumoniae* (see Fig 4). Moreover, just KO onto KO mice showed significantly increased pro- and anti-inflammatory cytokine liberation in their lungs by 72 h post-infection, when compared with WT onto KO mice (Fig 6G–6K). Together, these results demonstrate that expression of Mincle on alveolar immigrating leukocytes, most apparently neutrophils, is a critical determinant of lung protective immunity against pneumococcal pneumonia in mice.

## Discussion

The current study aimed to evaluate the role of C-type lectin Mincle in lung protective immunity against pneumococcal pneumonia in mice, using a focal pneumonia-inducing type 19F strain of *S*. *pneumoniae*. We here identified Glc-DAG to be the specific ligand by which *S*. *pneumoniae* is recognized by Mincle, which is supported by the following aspects: 1) Glc-DAG satisfies the common signature of Mincle ligand deduced from its known crystal structure [29, 30], 2) the Glc-DAG activity is eliminated in Mincle-deficient cells, demonstrating that Glc-DAG signals exclusively via Mincle, and 3) the observed *in vitro* activity of Glc-DAG is not due to contaminations such as e.g., endotoxin, as Glc-DAG has no activity on sensitive TLR4 reporter cells.

Diacylglycerol-containing glycolipids from *S*. *pneumoniae* were previously reported to be presented by CD1d for recognition by natural killer T cells via their invariant T cell receptor [31]. Our study shows for the first time that C-type lectin Mincle expressed by professional phagocytes is another receptor of the innate immune system to recognize the pneumococcus-derived diacylglycerol-containing glycolipid Glc-DAG. In fact, Glc-DAG represents a lipid anchor moiety of the TLR2 ligand lipoteichoic acid (LTA), one of the best characterized pathogen-associated molecular patterns of Gram-positive bacteria, such as *S*. *pneumoniae* and *S*. *aureus* [32, 33]. Though LTA itself does not act as a Mincle ligand [19], the current data show that Mincle can be activated through LTA fragments, i.e. its lipid anchor moiety Glc-DAG. These data add to our understanding how different moieties of the same ligand may be recognized by different classes of pattern recognition receptors.

Most of the currently available literature on the role of Mincle in infectious diseases relates to its role in fungal and mycobacterial infections [13, 15, 16, 21], whereas relatively little knowledge exists regarding its role in bacterial infections. Recently, a protective role of Mincle in bacterial pneumonia caused by Gram-negative *Klebsiella pneumoniae* was reported, where lack of Mincle resulted in defective neutrophil phagocytosis of *K*. *pneumoniae* [34]. In the current model of pneumococcal pneumonia, macrophage and neutrophil phagocytosis as well as ROS production and hence oxidative intracellular killing were not dependent on the presence of Mincle. Moreover, neutrophil-dependent NET formation reported to play a role in host defense against *K*. *pneumoniae* [34], according to previous reports, appears to be ineffective in antibacterial responses against *S*. *pneumoniae* [35]. More recent reports even demonstrate that NET formation may facilitate outgrowth of type 19F *S*. *pneumoniae* in a model of middle ear infection [36]. Based on currently available data, Mincle does not appear to induce direct effectors involved in antibacterial immunity against *S*. *pneumoniae*. Rather, we suggest that in pneumococcal pneumonia, the principal function of Mincle as ITAM-coupled receptor is to orchestrate immediate-early pro- and anti-inflammatory responses regulating lung protective immunity against pneumococci.

We found that Mincle is not protective in a model of IPD, consistent with earlier reports [28]. In contrast, Mincle was indispensable for protection against focal pneumonia. The observed role for Mincle in protective immunity against focal pneumonia but not IPD is most likely due to the different pathogenicity profiles, and thus clinical disease courses, of the employed non-invasive as compared to invasive *S*. *pneumoniae* serotypes used in the current study. Since highly invasive strains of *S*. *pneumoniae* such as the currently employed serotype 3 (A66.1) *S*. *pneumoniae* are known to cause early bacteremia and IPD in mice within 24 h [26], lung-directed antibacterial immunity mediated by Mincle-expressing phagocytes is expected to be more effective against non-invasive as compared to invasive serotypes of *S*. *pneumoniae* rapidly escaping lung innate immune surveillance. This view is supported by our observation in chimeric Mincle KO mice (carrying the hematopoietic system of WT mice) that were found to recruit Mincle-expressing neutrophils to the alveolar air space in response to pneumococcal infection, which was sufficient to normalize antibacterial and cytokine responses after pneumococcal challenge. These data demonstrate a critical role for Mincle expressing leukocytes, most apparently neutrophils, to contribute to regulation of protective immunity against focal pneumonia-causing *S*. *pneumoniae*.

Collectively, the current study provides novel informations about lung phagocyte recognition of *S*. *pneumoniae* by C-type lectin receptor Mincle, which critically contributes to lung protective immunity against this serious lung pathogen. The study may be relevant to the development of novel interventions to enhance lung phagocytic responses against this prototypic lung pathogen in a CLR specific manner.

## Materials and Methods

### Mice

WT C57BL/6J mice were purchased from Charles River (Sulzfeld, Germany). Mincle KO mice on a C57BL/6J background were generated as previously described [21] and were obtained from the Consortium for Functional Glycomics (CFG). Myd88-deficient mice on a C57BL6/J background were generated as described previously [37]. Sex- and age-matched mice (8–12 weeks of age) were used for experiments. Animals were handled according to institutional guidelines of the Central Animal Facility of Hannover School of Medicine. All animal experiments were approved by the Lower Saxony State Office for Consumer Protection and Food Safety.

### Reagents

Rat monoclonal anti-murine Mincle antibody (clone 4A9, IgG1κ) specifically recognizing Mincle but not MCL was generated as recently described [13]. Respective purified rat IgG1κ isotype control (clone R3-34) was purchased from BD Biosciences (Heidelberg, Germany). Anti-CD11b PE-Cy7 (clone M1/70), anti-Ly6G PE (clone 1A8), anti-MHC class II PE (clone 1G9), biotinylated IgG1 (clone RG11/39.4), allophycocyanin (APC)-labeled Annexin V, APC-labeled streptavidin, and propidium iodide (PI) were all obtained from BD Biosciences (Heidelberg, Germany). Anti-F4/80 FITC and anti-F4/80 APC (clone CI:A3-1) were obtained from Serotec (Düsseldorf, Germany). Anti-CD11c PE-Cy5.5 (clone N418) was purchased from Invitrogen (Darmstadt, Germany).

### Culture and quantification of *S*. *pneumoniae*

Serotype 19F and serotype 3 *S*. *pneumoniae* were grown in Todd-Hewitt broth (Oxoid, Basingstoke, UK) enriched with 20% FCS (Biochrom, Berlin, Germany) to mid-log phase. Quantification of pneumococci was done by plating ten-fold serial dilutions on sheep blood agar plates (BD Biosciences, Heidelberg, Germany) followed by incubation at 37°C, 5% CO_2_ for 18 h and subsequent determination of CFU, as described [27, 38, 39].

### Purification and characterization of Mincle-binding glycolipid from *S*. *pneumoniae*

For lipid extraction, bacteria were centrifuged at 2,400 g for 20 min. Lipids were extracted from bacterial pellets with chloroform-methanol-water (C:M:W, 6:3:1, v/v/v) overnight, and the C:M layer was collected after centrifugation. After drying up in an N_2_ evaporator, lipids were reconstituted in C:M (2:1, v/v) and resolved by HPLC column (Inertsil SIL-100A, 5 μm, 7.6 x 250 mm; GL Science) with C:M (9:1, v/v). Each sample fractionated with HPLC was dried up and dissolved with C:M (2:1, v/v). After HPLC, fractions were analyzed on high-performance thin-layer chromatography (HPTLC) plates (Silica gel 60; Merck) with C:M (9:1, v/v). Copper acetate was used for visualization of lipids. Those fractions providing GFP signals in NFAT-GFP reporter cell assays were further separated by scratching plates after TLC. Scratched lipids were extracted with C:M (2:1, v/v) again. Extracted Mincle ligand was subsequently analyzed with electrospray ionization time-of-flight mass spectrometry (ESI-TOF-MS) using a MicrOTOF II (Bruker Daltonics Inc., MA, USA). Glc-DAG was heated with 50 μl of 10% HCl/MeOH in a sealed tube at 80°C for 3 h. The reaction mixture was diluted with 0.5 ml of MeOH and extracted with n-hexane, and the n-hexane extract was concentrated *in vacuo* to give a mixture of fatty acid methylesters (FAMEs). The FAMEs were dissolved in acetone and subjected to gas chromatography-mass spectrometry (GC-MS) using a Shimadzu QP-5050A (Kyoto, Japan) equipped with a TC-1701 capillary column (GL science, Tokyo, Japan). The remaining MeOH layer was neutralized with Ag_2_CO_3_, and filtrated. The filtrate was dried in vacuo and then dissolved in 25 μl of pyridine, followed by addition of 25 μl of 1-(trimethylsilyl) imidazole. The reaction mixture was heated at 60°C for 20 min, and the TMS ether of glycoside was analyzed by GC-MS.

Synthesis of alpha-glucosyl-dimyristylglycerol (C14:0) and alpha-glucosyl-distearylglycerol (C18:0) was performed through the adaptation of literature reported methods [40, 41].

### Reporter cell assays

2B4-NFAT-GFP reporter cells expressing FcRγ only, or co-expressing FcRγ and Mincle were prepared as previously described [13]. Aliquots of lipid fractions (prepared as stock solutions in C:M (2:1) at 1 mg/ml) of *S*. *pneumoniae* were diluted in isopropanol and were then added to 96-well plates at various concentrations, followed by evaporation under a laminar flow hood. Subsequently, NFAT-GFP reporter cells were added to the wells (4 x 10^4^ cells/well), then incubated for 18 hours, and NFAT-GFP reporter transgene activity was determined by flow cytometry [13, 16, 22]. For TLR4 reporter cell assay, see online supplement.

### Stimulation of AM with Glc-DAG, TDM, or LTA

To elucidate the effect of highly purified pneumococcal Glc-DAG as compared to TDM or LTA on macrophage cytokine responses, 96-well plates were coated with Glc-DAG or TDM ligand (diluted in isopropanol) at 5 μg per well (TDM, 1 μg/well) followed by incubation of the plates at room temperature under a laminar flow hood until the solvent was completely evaporated. Then, 7.5 x 10^4^ murine or human AM were added and incubated for 24 h or 48 h, as indicated. Stimulation of murine alveolar macrophages with LTA (dissolved in sterile water) was done at 1 μg/ml for 24 h. Subsequently, proinflammatory cytokine release was measured in cell-free culture supernatants by enzyme-linked immunosorbent assay (ELISA).

### Infection of mice with *S*. *pneumoniae*

WT and Mincle KO mice were anesthetized with xylazine (5 mg/kg of body weight) (Bayer, Leverkusen, Germany) and ketamine (75 mg/kg) (Albrecht, Aulendorf, Germany) and then intubated orotracheally (o.t.) with a 29-gauge Abbocath catheter (Abbott, Wiesbaden, Germany) as described recently [42, 43]. Subsequently, mice were o.t. infected with either highly invasive serotype 3 (A66.1), or focal pneumonia-causing serotype 19F (EF3030) *S*. *pneumoniae* or mock-infected in a volume of 50 μl PBS, as indicated. After infection, mice were returned to their cages and monitored daily for disease symptoms. Survival of S. *pneumonia*e-infected mice was recorded daily for 6 (serotype 3 *S*. *pneumoniae*) or 8 days (serotype 19F *S*. *pneumoniae*).

### Bronchoalveolar lavage and quantification of leukocyte subsets

Bronchoalveolar lavage of mice was performed as outlined recently [23, 27, 42] and in the online supplement. Healthy volunteers and patients with severe pneumococcal pneumonia (n = 3 each) requiring mechanical ventilation underwent standard BAL procedure as previously described [44]. The diagnostic criteria for severe PN were characteristic chest X-rays, fever, dyspnea, and microbiological identification of pathogens in the lower respiratory tract as well as positive pneumococcal urinary antigen test (PUAT). Briefly, fiber-optic bronchoscopy was performed, and 20 ml aliquots of sterile saline were instilled into a subsegmental bronchus of the middle lobe or the lingula and aspirated by gentle suction. The recovered lavage samples were filtered through sterile gauze to remove mucus and immediately placed on ice. BAL fluid neutrophils comprised > 97% of total BAL fluid cells in pneumococcal pneumonia patients, while in healthy volunteers, resident alveolar macrophages comprised the majority of cellular constituents in BAL fluids (>95%), as analyzed by microscopic examination of Pappenheim-stained cytospin preparations. For isolation of peripheral blood neutrophils, heparinized peripheral blood (20 ml) was collected from patients with pneumococcal pneumonia and subjected to Ficoll-based density gradient centrifugation, resulting in purities of peripheral blood neutrophils of >95%, as previously described [45]. Written informed consent was received from healthy volunteers and patients with pneumococcal pneumonia or their closest relatives prior to BAL.

### Determination of bacterial loads in BAL fluid and lung tissue of *S*. *pneumoniae* infected mice

Bacterial loads were determined as outlined elsewhere [27] and in the online supplement.

### Immunophenotypic analysis of Mincle expression on lung leukocyte subsets

FACS analysis of Mincle expression on the various leukocyte subsets was performed as outlined recently [22, 23], and described in the online supplement.

### Analysis of necrosis induction in AM

Analysis of apoptosis/necrosis induction in AM collected from WT and Mincle KO mice infected with serotype 19F *S*. *pneumoniae* was done as described recently [27, 46], and in the online supplement.

### Phagocytosis and bacterial killing assay

Phagocytosis and bacterial killing were analyzed as described recently [27, 46]. Briefly, alveolar macrophages recovered by BAL from untreated WT and Mincle KO mice were seeded at 2 x 10^5^ cells per well into cell culture plates. After adherence (30 min.), AM were washed and infected with *S*. *pneumoniae* at MOI 50 for 30 min. in RPMI/10% FCS/1% glutamine at 37°C/5% CO_2_. Neutrophils were purified from bone marrow cells of WT and Mincle KO mice [22] and were infected with *S*. *pneumoniae* at MOI 50 in polypropylene tubes for 30 min. in RPMI/10% FCS/1% glutamine at 37°C/5% CO_2_. Subsequently, non-phagocytosed bacteria were removed by three washing steps, and residual extracellular pneumococci were killed by short incubation (10 min.) in RPMI/10% FCS/1% glutamine/20 μg/ml gentamicin (Sigma, Deisenhofen, Germany), after which neutrophils were seeded at 2 x 10^5^ cells per well into cell culture plates. After 30 min. of infection, cells were either lysed for determination of pneumococcal uptake, or were incubated for another 60 and 90 min. followed by cell lysis in 0.1% saponin in HBSS to release intracellular pneumococci and determination of CFU by plating ten-fold serial dilutions of cell lysates on sheep blood agar plates and subsequent incubation of plates at 37°C/5% CO_2_ for 18 h.

### Analysis of burst induction in resident AM and bone marrow-derived neutrophils

96 well plates were coated with vehicle (isopropanol) or Glc-DAG (5 μg/well in isopropanol) followed by evaporation of the solvent. AM were harvested from the lungs of WT and Mincle KO mice by BAL. Bone marrow-derived neutrophils were purified according to recently published protocols [22]. Resident AM and neutrophils were added to Glc-DAG coated wells at 7.5x10^4^ cells/well. After addition of 2 mM luminol (Sigma, Deisenhofen, Germany), burst induction was analyzed at 37°C after 3 h of AM or neutrophil exposure to vehicle, or Glc-DAG and is expressed as relative light units (RLU) using a luminescence reader (BioTec Instruments, Bad Friedrichshall, Germany, KC4 software). As a positive control, AM and neutrophils were exposed to *S*. *pneumoniae* (Spn, MOI 5), and RLU were measured 3 h later.

### Real-time RT-PCR

Mincle gene expression was determined in lung tissue or flow-sorted AM and neutrophils purified from the lungs of mice by high-speed cell sorting (BD FACSAria II, BD Biosciences, Heidelberg, Germany), as recently described [23, 27, 42], or in BAL fluid neutrophils from patients with pneumococcal pneumonia. For further details, see online supplement.

### Quantification of proinflammatory cytokines

See online supplement.

### Lung histopathology

Lung histopathology was assessed as described in the online supplement.

### Statistics

All data are given as mean ± SD and were analyzed using GraphPad Prism Software. Differences between treatment groups were analyzed by Mann-Whitney U test. Survival curves were compared by log-rank test. Unpaired t-test was used to determine differences between Glc-DAG treated and vehicle treated cells *in vitro*. Statistically significant differences between treatment groups were assumed when *P* values were < 0.05.

### Ethics statement

Animal experiments were approved by the Lower Saxony State Office for Consumer Protection and Food Safety (LAVES) (permission numbers 13/1063 and 15/1743), and followed the European Council Directive 2010/63/EU as well as the German Animal Welfare Act.

## Methods

### Bronchoalveolar lavage and quantification of leukocyte subsets

At indicated time points, *S*. *pneumoniae*-infected WT and Mincle KO mice were euthanized with an overdose of isoflurane (Baxter, Unterschleissheim, Germany) and subjected to bronchoalveolar lavage. Briefly, the trachea was cannulated with a shortened 20-gauge needle and 300 μl aliquots of cold PBS (Biochrom) supplemented with EDTA (Versen, Biochrom, Berlin, Germany) were instilled, followed by careful aspiration, until a volume of 1.5 ml was collected. BAL was then continued until an additional BAL fluid (BALF) volume of 4.5 ml was obtained. BAL fluids were centrifuged at 1,400 rpm at 4°C for 9 min and cell pellets were resuspended in RPMI 1640 supplemented with 10% FCS and total cell numbers of BAL fluid leukocytes were determined. BAL fluid leukocyte subsets were differentiated on Pappenheim-stained cytospin preparations based on overall morphological criteria, including cell size and shape of nuclei, and were then quantified by multiplication of respective percent values with total BAL cell counts.

### Determination of bacterial loads in BAL fluid and lung tissue of *S*. *pneumoniae* infected mice

After BAL was performed, individual lung lobes were removed and dissected into small pieces, and were then homogenized in 2 ml of HBSS using a tissue homogenizer (IKA, Staufen, Germany). Lung tissue homogenates were then filtered through a 100 μm cell strainer (BD Bioscience, Heidelberg, Germany). Bacterial loads in BAL fluids and lung tissue homogenates were determined by plating ten-fold serial dilutions of lung tissue homogenates on sheep blood agar plates, followed by incubation at 37°C/5% CO_2_ for 18 h and subsequent determination of total CFU counts.

### Generation of chimeric Mincle KO mice

Mincle KO mice received whole body irradiation (8 Gy), followed by transplantation with bone marrow cells (10^7^ cells/mouse i.v.) from either WT mice, or Mincle KO mice serving as transplantation controls. Seven weeks later, mice were infected orotracheally with 1x10^7^ CFU/mouse of type 19 *S*. *pneumoniae*, followed by determination of bacterial loads in BAL fluids and lung tissue of mice. In selected experiments, Mincle expression was analyzed on the cell surface of neutrophils contained in bronchoalveolar lavage fluid from *S*. *pneumoniae*-infected (24 h) chimeric Mincle KO mice reconstituted with the hematopoietic system of WT or Mincle KO mice.

In selected experiments, we verified that Mincle deficiency did not impact on hematopoietic engraftment efficacy in chimeric WT (CD45.1^pos^) onto Mincle KO mice, and in chimeric Mincle KO (CD45.2^pos^) onto WT (CD45.1^pos^) mice at 6 weeks post-transplantation, which was always found to be >90%.

### Immunophenotypic analysis of leukocyte subsets in BAL fluid and lung tissue

Lungs were perfused in situ via the right ventricle with HBSS. Then, lung lobes were collected, teased into small pieces and digested in RPMI/collagenase A (5 mg/ml) and DNase I (1 mg/ml) (Roche, IN) for 90 min at 37°C. Tissue digestion was stopped by addition of RPMI/10% FCS, followed by filtration of digested lung tissue through 40 μm cell strainers (BD Falcon). CD11c-positive lung leukocyte subsets were enriched by magnetic cell purification (Miltenyi, Bergisch Gladbach, Germany). Aliquots of lung tissue digests were employed for analysis of Mincle expression on lung neutrophils. BAL and lung leukocyte subsets were immunophenotyped as described in detail recently [27, 46, 47]. Briefly, after pre-incubation with Octagam (Octapharma, Langenfeld, Germany), 2–5 x 10^5^ cells were incubated with fluorochrome-labeled mAbs with specificity for F4/80, CD11b, CD11c, Ly6G, and MHC II for 20 min. at 4°C. Subsequently, cells were washed twice with FACS buffer and subjected to FACS analysis of their cell surface antigen (Ag) expression profiles. Resident AM contained in BAL fluids were characterized according to their green autofluorescence and F4/80^pos^, CD11b^neg^, CD11c^pos^ antigen expression profile, and green autofluorescent, inflammatory recruited alveolar ‘exudate’ macrophages (ExMacs) were characterized by their F4/80^pos^, CD11b^pos^, CD11c^pos^ Ag expression profile (see S1 Fig). Note that gating of alveolar recruited exudate macrophages at 24 h post-infection may to a small extent (5–10%) include inflammatory activated resident alveolar macrophages, according to previous reports [48].

Neutrophils in BAL and lung tissue of WT and chimeric mice were characterized according to their CD11c^neg^, CD11b^pos^, Ly6G^pos^ antigen expression profile. Subsequently, appropriately gated resident AM and ExMacs in BAL fluids, as well as lung and BAL neutrophils were analyzed for their respective Mincle cell surface expression profiles using anti-Mincle Ab clone 4A9.

### Analysis of necrosis induction in AM

Analysis of apoptosis/necrosis induction in AM collected from WT and Mincle KO mice after infection with serotype 19 *S*. *pneumoniae* was done by incubating BAL cells with APC-labeled annexin V (apoptosis marker) in the presence of propidium iodide for 15 min at room temperature according to the manufacturer’s instructions (BD Biosciences), followed by FACS-based determination of annexin V^neg^/propidium iodide^pos^ necrotic macrophages in the fraction of F4/80-positive AM.

### Real-time RT-PCR

Total RNA from lung tissue or cell specimen was isolated using RNeasy Micro kit (Qiagen, Hilden, Germany), following the manufacturer’s instructions. For cDNA synthesis, 100 ng of purified total cellular RNA was reverse transcribed and quantitative real-time RT-PCR was performed on an ABI 7300 real-time PCR System (Applied Biosystems, Warrington, United Kingdom) using SYBR Green dye (Eurogentec, Seraing, Belgium), as recently described. Murine Mincle specific primers were forward primer 5’-‘TCAACCAAATCGCCTGCAT-3’, and reverse primer 5’-GAGGCCCCGGCTATCGT-3‘. Primers were designed using Primer Express software (Applied Biosystems, Warrington, UK), based on the gene sequence data retrieved from GenBank. For normalization, murine β-actin (forward primer, 5’-CCACAGCTGAGAGGGAAATC-3’, and reverse primer 5’-TCTCCAGGGAGGAAGAGG AT-3’) was used as the housekeeping gene. Human Mincle specific primers were forward 5’-CATTTCGCATCTTTCAAACCTGTG-3’, and reverse 5’ ATTCCCAGTTCAATGGA CAACAATT-3’. Human β-actin specific primers were forward 5’-GCCA CGGC TGC TTCCA-3’ and reverse 5’-GAACCGCTCATTGCCATTG-3’. Mean fold changes were calculated using the 2^-ΔΔCt^ method [49].

### Lung histopathology

WT and Mincle KO mice were euthanized and non-lavaged lungs were inflated in situ with PBS-buffered formaldehyde solution (4.5%, pH 7, Roth, Deisenhofen, Germany), and were then removed en bloc and fixed in PBS-buffered formaldehyde solution at room temperature for at least 24 h. Following automated dehydration, routine paraffin embedding, lung tissue sections (3 μm) were prepared and then stained with hematoxylin/eosin (HE). Subsequently, the percentage of inflamed lung tissue from each lung lobe of the left and right lung per mouse was determined in *S*. *pneumoniae*-infected WT and Mincle KO mice as mean values of affected lung tissue using a Zeiss Axiovert 200 M microscope (Carl Zeiss, Wetzlar, Germany). Lung histology assessment was performed under blinded conditions.

### TLR4 reporter cell assay and stimulation of bone marrow-derived phagocyte subsets from WT and Myd88 KO mice with Glc-DAG

To exclude that Glc-DAG contained any lipopolysaccharide (LPS) or other contaminants signaling via TLR4 or Myd88, control experiments were performed in TLR4 reporter cells and bone marrow-derived phagocytes from WT and Myd88 KO mice. HEK-Blue mTLR4 reporter cells and TLR4 deficient HEK-Blue Null1-v cells (both InvivoGen) were employed to exclude any TLR4 ligand activity in our Glc-DAG preparations, while bone marrow-derived phagocytes from WT and Myd88-deficient mice generated as recently described [22] were used to exclude any contaminations in Glc-DAG preparations signaling via Myd88-dependent pathways. Lipopolysaccharide (LPS, L4516 derived from *Escherichia coli* 0127:B8, Sigma) serving as prototypical TLR4 ligand was used as positive control. HEK-blue cells stably express secreted embryonic alkaline phosphatase (SEAP) gene inducible by NF-κB. Cells were seeded in a 96-well plate (3x10^4^ cells/well in 100 μl) with or without stimulants (LPS at 0.1 ng/ml or Glc-DAG at 5 μg per well) and were then incubated overnight. Medium samples (5 μL) were then mixed with QUANTI-Blue (InvivoGen) medium (45 μL) and incubated at 37°C for 3 hours. Levels of SEAP in the medium were determined by measurement of OD values at 630 nm using a Multiskan microplate reader (Thermo).

### Quantification of proinflammatory cytokines

Pro- and anti-inflammatory cytokines were measured using commercially available ELISA kits for TNF-α (detection limit: 11 pg/ml), IL1-β (detection limit: 12 pg/ml), KC (6 pg/ml), IL-10 (detection limit: 16 pg/ml), or IL-1ra (32 pg/ml), according to the manufacturer’s instructions (R&D Systems, Wiesbaden, Germany).

## Supporting Information

S1 Fig Flow cytometric analysis of resident alveolar macrophages and alveolar exudate macrophages in BAL fluids of WT and Mincle KO mice. WT and Mincle KO mice were either left uninfected or were infected with type 19F *S*. *pneumoniae* (10^7^ CFU/mouse in 50 μl PBS). Twenty-four hours after infection, mice were subjected to BAL, and alveolar macrophages (AM) were gated according to their FSC-A/SSC-A characteristics (P1 in A, D, G, J), followed by sub-gating according to their FSC-A/F4/80 characteristics (P2 in B, E, H, K). Subsequently, resident AM in BAL fluids were identified according to their CD11c^pos^/CD11b^neg^ phenotype, while exudate macrophages (ExMacs) in BAL fluids exhibited a CD11c^pos^/CD11b^pos^ immune-phenotype (illustrated in C, F, I, L). The dot plots are representative of n = 8 FACS analyses per treatment group with similar results.
(TIF)

S2 Fig Biochemical characterization of the putative Mincle ligand of *S*. *pneumoniae*. (A) Electrospray ionization-mass spectrometry (ESI-MS) spectrum of the purified glycolipid showing four prominent peaks with mass-to-charge (m/z) ratios = 697.4875, 725.5135, 751.5316, and 779.5608. (B,C) Gas chromatography-mass spectrometry (GC-MS) spectrum of fatty acid methyl esters (FAMEs), including methyl-palmitate with retention time (rt) = 14.48, corresponding to m/z = 270 (M^+^), as well as further fragment peaks with m/z = 239, 227, 199, 143, and 87. (D) GC spectrum of trimethylsilyl (TMS) ether of methyl glycoside, Tri-*O*-TMS glycerol with rt = 6.3 min, 1-OMe-tetra-*O*-TMS glucose with rt = 13.9 and 14.1 min. These rt were identical with the standard samples. (E) The common structure of glucosyl-diacylglycerol (Glc-DAG) of *S*. *pneumoniae*.
(PDF)

S3 Fig Effect of Glc-DAG stimulation on TLR4 reporter cells and Myd88-deficient mouse phagocytes. (A) TLR4-sufficient and -deficient reporter cells were stimulated with either LPS (0.1 ng/ml) or Glc-DAG (5 μg/well) for 20 hours, followed by determination of secreted embryonic alkaline phosphatase (SEAP) activity in cell culture supernatants expressed as optical density (OD) at 630 nm. Note that Glc-DAG did not activate TLR4 reporter cells. The data are expressed as mean ± SD of duplicate determinations, and are representative of two independent experiments. (B,C) Bone marrow-derived phagocytes from WT and Myd88-deficient mice were either left untreated (Control), or were stimulated with LPS (100 ng/ml) or Glc-DAG (5 μg/well) for 48 h, followed by analysis of TNF-α (B) and MIP-2 (C) cytokine levels in cell-free culture supernatants by ELISA. Note that Glc-DAG induced a similar cytokine response in WT and Myd88-deficient cells, whereas LPS induced a cytokine response in wild-type cells only. The data are expressed as mean ± SD of triplicate determinations, and are representative of two independent experiments. **p at least <0.01, relative to control (A) or WT (B,C).
(TIF)

S4 Fig Respiratory burst induction in resident alveolar macrophages and bone marrow-derived neutrophils from WT and Mincle KO mice after stimulation with Glc-DAG purified from *S*. *pneumoniae*. Resident AM were collected by bronchoalveolar lavage from the lungs of untreated WT and Mincle KO mice, and bone marrow-derived neutrophils were purified from respective mice as described in Materials and Methods. rAM and neutrophils were seeded into vehicle (isopropanol) or Glc-DAG (5 μg/well) coated 96-well plates (7.5 x 10^4^ cells/well). (A, B) Oxidative burst induction in rAM at 3 h post-exposure to vehicle, Glc-DAG (5 μg/well), or *S*. *pneumoniae* (Spn, MOI 5) (A), or oxidative burst induction in neutrophils at 3 h post-exposure to vehicle, Glc-DAG (5 μg/well), or *S*. *pneumoniae* (Spn, MOI 5) (B). Results are shown as mean ± SD of n = 3–4 determinations per treatment condition and experimental group and are representative of two independent experiments. **p at least <0.01 relative to vehicle controls. RLU, relative light unit.
(TIF)

S5 Fig Alveolar leukocyte recruitment in WT and Mincle KO mice infected with *S*. *pneumoniae*. WT and Mincle KO mice were infected with *S*. *pneumoniae* (10^7^ CFU/mouse), and at indicated time points post-infection, mice were subjected to BAL and numbers of AM (A), or newly immigrated BAL exudate macrophages (B), or BAL neutrophils (C) were determined. Data are shown as mean ± SD of n = 5–8 mice per time point and treatment group (except at 12 h, where n = 4 mice per time point and treatment group). Data are representative of two independent experiments with similar results. **p<0.01 relative to WT mice.
(TIF)

S6 Fig Effect of Mincle on phagocytosis and killing of *S pneumoniae* by resident alveolar macrophages and bone marrow-derived neutrophils *in vitro*. Resident AM (A) (rAM, 2x10^5^ cells/well) and bone marrow-derived neutrophils (B) (2x10^5^ cells/well) were infected with *S*. *pneumoniae* (MOI 50) for 30 minutes, as described in Materials and Methods, for uptake of bacteria during this time interval. After washing and short-term gentamicin treatment to kill extracellular bacteria, cells were further incubated for another 60 and 90 min. for analysis of bacterial killing at 90 and 120 minutes post-infection. Data are shown as mean ± SD of quadruplicate determinations per time point and treatment group. The experiment was performed twice with similar results.
(TIF)

S1 Table Theoretical molecular weight equivalent to each m/z peak. Elemental composition deduced from high resolution ESI-TOF-MS and structural assignment of fatty acids binding to glycerol backbone based on GC-MS after acid hydrolysis.
(PDF)
