# Supplementary figures and images for: C-type Lectin Mincle Recognizes Glucosyl-diacylglycerol of Streptococcus pneumoniae and Plays a Protective Role in Pneumococcal Pneumonia
Source: PLoS Pathog. 2016 Dec 6;12(12):e1006038. doi: 10.1371/journal.ppat.1006038 (PMC5140071; doi:10.1371/journal.ppat.1006038)

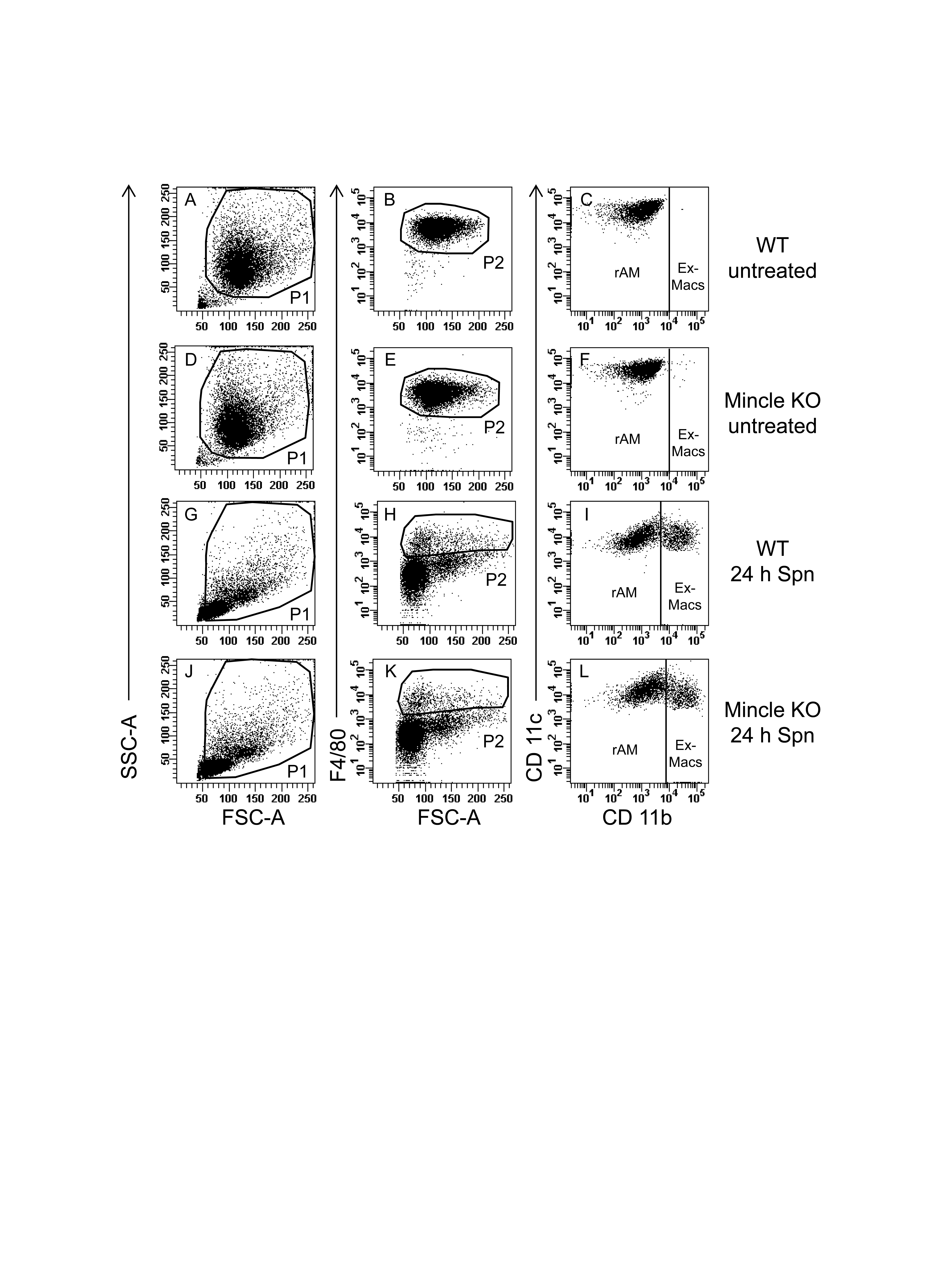

Supplement: S1 Fig — WT and Mincle KO mice were either left uninfected or were infected with type 19F S. pneumoniae (107 CFU/mouse in 50 μl PBS). Twenty-four hours after infection, mice were subjected to BAL, and alveolar macrophages (AM) were gated according to their FSC-A/SSC-A characteristics (P1 in A, D, G, J), followed by sub-gating according to their FSC-A/F4/80 characteristics (P2 in B, E, H, K). Subsequently, resident AM in BAL fluids were identified according to their CD11cpos/CD11bneg phenotype, while exudate macrophages (ExMacs) in BAL fluids exhibited a CD11cpos/CD11bpos immune-phenotype (illustrated in C, F, I, L). The dot plots are representative of n = 8 FACS analyses per treatment group with similar results. (TIF) [file ppat.1006038.s001.tif]

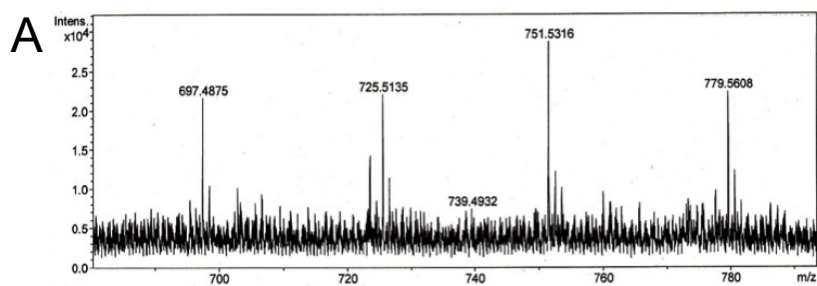

Methyl palmitate  
rt=14.5 min.

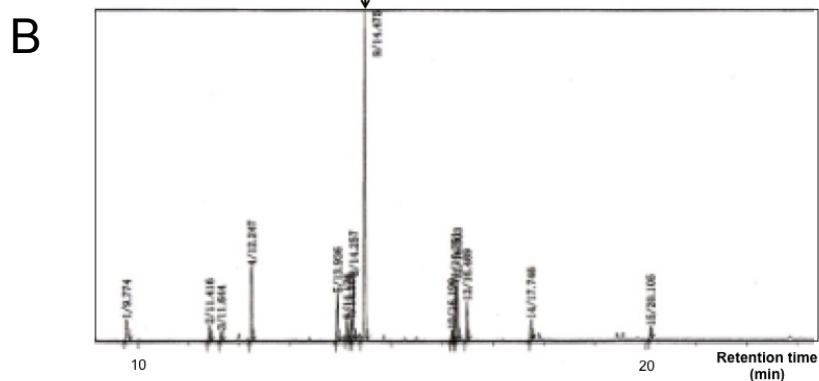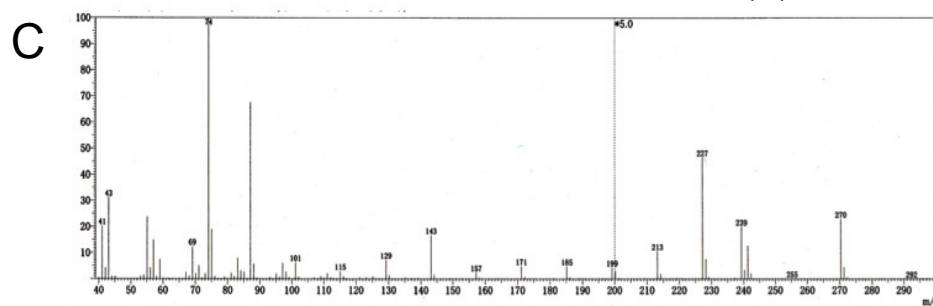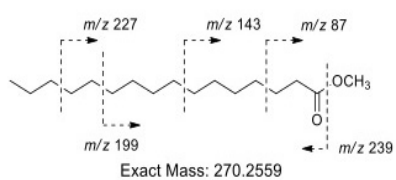

**D**

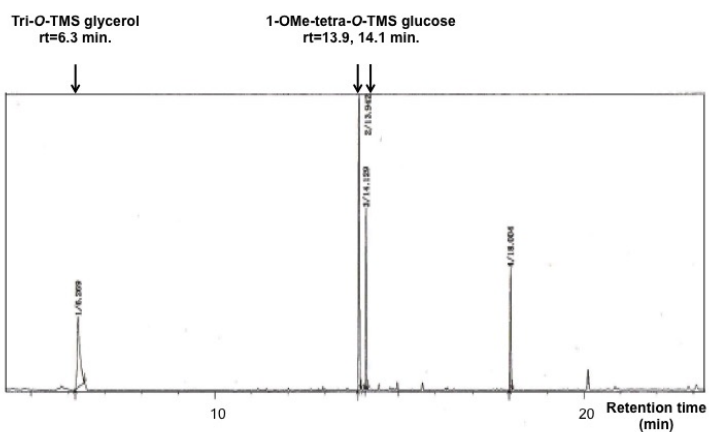

**E**

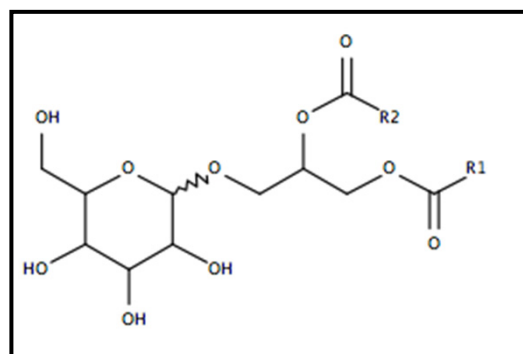

Supplement: S2 Fig — (A) Electrospray ionization-mass spectrometry (ESI-MS) spectrum of the purified glycolipid showing four prominent peaks with mass-to-charge (m/z) ratios = 697.4875, 725.5135, 751.5316, and 779.5608. (B,C) Gas chromatography-mass spectrometry (GC-MS) spectrum of fatty acid methyl esters (FAMEs), including methyl-palmitate with retention time (rt) = 14.48, corresponding to m/z = 270 (M+), as well as further fragment peaks with m/z = 239, 227, 199, 143, and 87. (D) GC spectrum of trimethylsilyl (TMS) ether of methyl glycoside, Tri-O-TMS glycerol with rt = 6.3 min, 1-OMe-tetra-O-TMS glucose with rt = 13.9 and 14.1 min. These rt were identical with the standard samples. (E) The common structure of glucosyl-diacylglycerol (Glc-DAG) of S. pneumoniae. (PDF) [file ppat.1006038.s002.pdf]
