# Supplementary material for: C-type Lectin Mincle Recognizes Glucosyl-diacylglycerol of Streptococcus pneumoniae and Plays a Protective Role in Pneumococcal Pneumonia
Source: PLoS Pathog. 2016 Dec 6;12(12):e1006038. doi: 10.1371/journal.ppat.1006038 (PMC5140071; doi:10.1371/journal.ppat.1006038)
Supplement: S1 Table — Elemental composition deduced from high resolution ESI-TOF-MS and structural assignment of fatty acids binding to glycerol backbone based on GC-MS after acid hydrolysis. (PDF) [file ppat.1006038.s007.pdf]

Measured Mass (Da)	Elemental composition	Theoretical Mass (Da)	Structures
697.4875	C37H70O10Na	697.4861	(16:0/12.0), (14:0/14:0)
725.5135	C39H74O10Na	725.5174	(16:0/14:0), (12:0/18:0)
751.5316	C41H76O10Na	751.5331	(16:0/16:1)
779.5608	C43H80O10Na	779.5644	(16:0/18:1)
